# Recent advances in N-heterocyclic carbene (NHC)-catalysed benzoin reactions

**DOI:** 10.3762/bjoc.12.47

**Published:** 2016-03-09

**Authors:** Rajeev S Menon, Akkattu T Biju, Vijay Nair

**Affiliations:** 1 Department of Chemistry, Central University of Haryana, Mahendergarh, Haryana-123 029, India; 2 Organic Chemistry Division, National Chemical Laboratory (CSIR), Dr. Homi Bhabha Road, Pune 411 008, India; 3 Organic Chemistry Section, CSIR-National Institute for Interdisciplinary Science and Technology,Trivandrum 695 019, India.; Fax: +91 471 2491712; Tel: +91 471 2490406

**Keywords:** acyloin reaction, benzoin reaction, N-heterocyclic carbenes, organocatalysis, umpolung

## Abstract

N-Heterocyclic carbenes (NHCs) have emerged as a powerful class of organocatalysts that mediate a variety of organic transformations. The Benzoin reaction constitutes one of the earliest known carbon–carbon bond-forming reactions catalysed by NHCs. The rapid growth of NHC catalysis in general has resulted in the development of a variety of benzoin and benzoin-type reactions. An overview of such NHC*-*catalysed benzoin reactions is presented.

## Introduction

The benzoin reaction (or condensation) is named after the product it furnishes via a catalytic assembly of two molecules of aromatic aldehydes. One molecule of the aldehyde functions as an acyl anion and the other as a carbonyl electrophile to afford α-hydroxy ketones (benzoins). It is a 100% atom-economic process wherein a new stereocentre is produced. The reaction is sometimes referred to as acyloin condensation to encompass reactions of aliphatic aldehydes. The assembly of two molecules of the same aldehyde is known as homo-benzoin reaction and that of two different aldehydes is known as crossed benzoin reaction. Mechanistically the reaction involves polarity reversal (umpolung) of one aldehyde to generate an acyl anion equivalent and this event is mediated by the catalyst. Alkali metal cyanides and N-heterocyclic carbenes (NHCs) are the two main classes of catalysts that are known to mediate benzoin reactions. This review focuses on the recent advancements made in the area of NHC-catalysed benzoin reactions.

Historically, the first benzoin reaction was reported by Wöhler and Liebig in 1832. They discovered that the cyanide anion can catalyze the union of two molecules of aromatic aldehydes to afford α-hydroxy ketones [1]. More than a century later, a thiazolium salt-catalysed benzoin reaction was reported by Ukai [2]. This may be regarded as an early example of organocatalysis using an azolium salt. Breslow postulated in 1958 a mechanistic rationale for the thiazolium salt-catalysed benzoin reaction [3]. He depicted the catalytically active species as a thiazolium zwitterion (the resonance structure of an NHC) and proposed that the reaction proceeds via an enaminol intermediate. The latter is now popularly known as ‘Breslow intermediate’. This seminal discovery by Breslow paved the way for further developments in the area of carbene catalysis. Almost three decades later Bertrand and co-workers proved the existence of carbenes as catalytically active species in the benzoin reaction, with the synthesis of a stable phosphinocarbene [4]. Arduengo and co-workers isolated and characterised a stable NHC in 1991 [5]. These two reports on the isolation of NHCs implied that they are more stable and robust than previously considered. Subsequent years witnessed a renewal of interest in NHCs and a flurry of reports, mainly focusing on their catalytic activity, appeared in the literature [6–7].

**Scheme 1:**
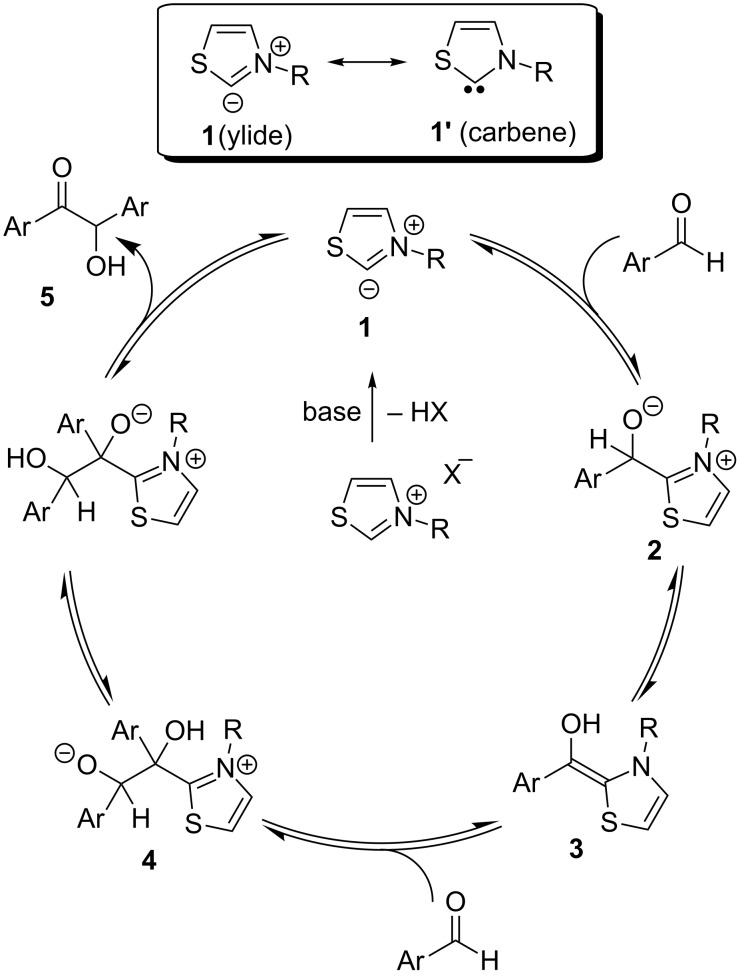
Breslow’s proposal on the mechanism of the benzoin condensation.

The original mechanistic proposal by Breslow for the thiazolium salt-catalysed benzoin reaction can be delineated as follows (Scheme 1) [3]. Lapworth had suggested how the cyanide anion functions first as a nucleophile and then as a leaving group in cyanide-catalysed benzoin reactions [8]. Analogously, Breslow invoked the generation of a nucleophilic thiazolylidene species **1** via deprotonation of the thiazolium salt by base. The ylide **1** may also be represented as its resonance structure **1’** (carbene). Nucleophilic addition of **1** to aromatic aldehyde generates the tetrahedral intermediate **2**. The latter then undergoes a proton shift to furnish an enaminol derivative **3**. The aldehyde carbonyl carbon has now transformed into a nucleophilic entity by virtue of conjugation to the nitrogen and sulfur lone pairs. This acyl anion equivalent **3** is known as the "Breslow intermediate". Its reaction with another molecule of aldehyde leads to the formation of an alkoxide intermediate **4**. Proton transfer and subsequent release of thiazolylidene **1** affords the final product, the α-hydroxy ketone **5**. Breslow demonstrated that imidazolium-derived ylides also catalysed benzoin reactions. In most of the cases, the NHC-catalysed formation of benzoin from aldehydes is reversible in nature.

In the following sections, detailed discussions on various types of benzoin reactions catalysed by NHCs are presented. In general, thiazolium salt-derived NHCs have found widespread application as catalysts for benzoin reactions, whereas triazolium-derived NHCs have emerged as popular catalysts for enantioselective benzoin transformations.

## Review

### Homo-benzoin reactions

The homo-benzoin condensation constitutes an overall catalytic dimerization of an aldehyde wherein the acyl anion derived from one molecule adds to another molecule of the aldehyde. (It may be noted that the term ‘homo’ implies the reaction between two molecules of the same aldehyde. It should not be misconstrued as a ‘homologous’ benzoin reaction). Benzoin reactions are reversible in basic medium and homo-benzoin products are often isolated as byproducts in other NHC-mediated reactions of aldehydes. The absence of chemoselectivity issues makes homo-benzoin reactions less challenging when compared to the cross-benzoin variant. NHC-mediated aerial oxidation of aldehydes to the corresponding carboxylic acids could compete with homo-benzoin reactions, but can be limited by careful exclusion of oxygen from the reaction mixture. A few recent reports of homo-benzoin reactions are discussed in the following passages.

**Scheme 2:**
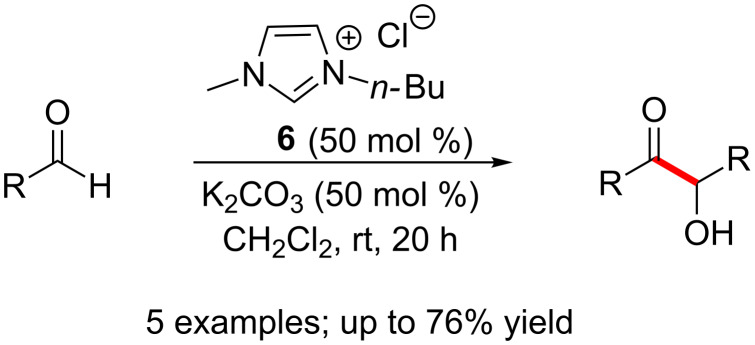
Imidazolium carbene-catalysed homo-benzoin condensation.

Stetter’s report in 1976 of thiazolium salt-catalysed benzoin reactions may be regarded as the first report of an NHC-catalysed benzoin reaction on a synthetically useful scale [9]. Much later, in 2005, Xu and Xia used *N*-alkyl-substituted imidazolium carbene **6** to efficiently promote benzoin reactions. Although a high catalyst loading (50 mol %) was required, the reactions could be run at mild conditions. It was observed that neutral and electron rich aromatic aldehydes afford good yields of benzoin products whereas electron deficient aromatic aldehydes and aliphatic aldehydes reacted sluggishly (Scheme 2) [10].

**Scheme 3:**
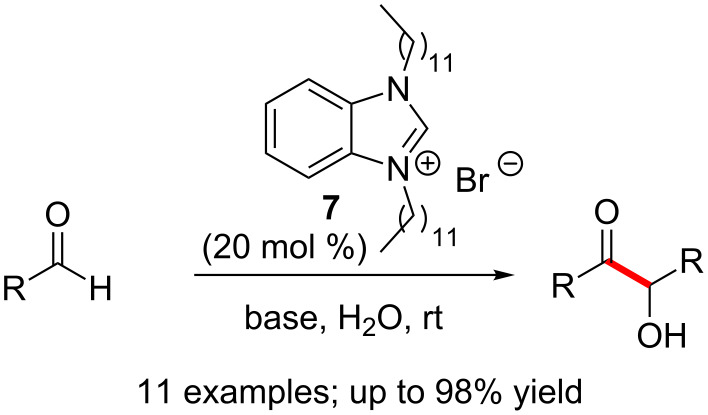
Homo-benzoin condensation in aqueous medium.

The easily accessible NHC precatalyst **7** endowed with long aliphatic side chains was used by Iwamoto and co-workers for promoting benzoin reactions in aqueous medium. The improved reactivity was attributed to the formation of micelles from the hydrophobic alkyl chains of the catalyst in aqueous medium. The reaction proceeded well with various aromatic and heteroaromatic aldehydes (Scheme 3) [11].

**Scheme 4:**
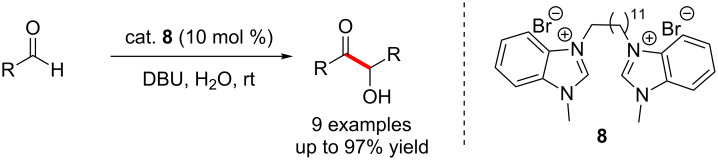
Homobenzoin condensation catalysed by bis(benzimidazolium) salt **8**.

Subsequently, the same group disclosed the application of bis(benzimidazolium) precursor **8** as a more efficient catalyst for the benzoin condensation in aqueous medium. Here, the NHC precatalyst incorporated a long aliphatic bridge between the two imidazolium entities. The aggregation of these units creates a hydrophobic environment in which the two aromatic aldehydes are subjected to catalysis (Scheme 4) [12].

### Asymmetric homo-benzoin reactions

Much of the progress in the area of NHC-catalysed asymmetric benzoin reactions has been covered in two excellent reviews [6–7]. Some additional recent examples are discussed below.

**Scheme 5:**
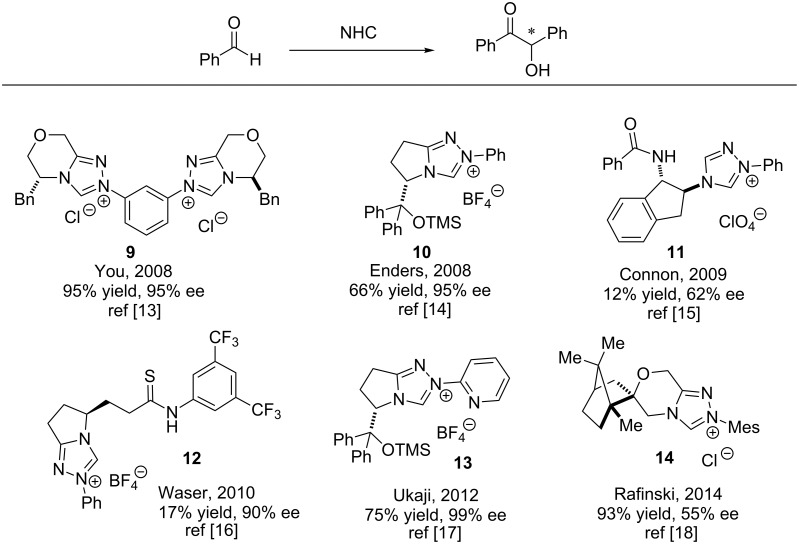
List of assorted chiral NHC-catalysts used for asymmetric homobenzoin condensation.

A selected list of chiral NHC catalysts that have been explored for mediating asymmetric benzoin reactions is presented in Scheme 5. The bis-triazolium catalyst **9** developed by You promoted asymmetric benzoin reactions in 95% ee [13]. Enders developed the pyroglutamic acid-derived triazolium salt **10** which mediated benzoin reactions in similar enantioselectivities [14].

The chiral triazolium catalyst **11** transfers chiral information to the benzoin products by engaging in hydrogen-bonding interactions [15]. Waser’s chiral bifunctional (thio)urea NHC **12** also relies on hydrogen bonding to mediate asymmetric benzoin reactions [16]. A 2-pyrdiyl appendage distinguishes Ukaji’s chiral triazolium catalyst **13** from similar salts [17]. Spirocyclic (1*R*)-camphor-derived triazolium salt **14**developed by Rafiński also successfully catalysed asymmetric benzoin condensations [18].

**Scheme 6:**
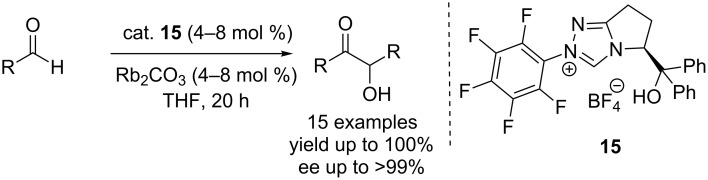
A rigid bicyclic triazole precatalyst **15** in an efficient enantioselective benzoin reaction.

The pentafluorophenyltriazolium catalyst **15** featured in the most efficient asymmetric benzoin reaction reported so far. Inoue and co-workers found that it promotes homocoupling of benzaldehyde at a low loading (4 mol %) to afford benzoin in 90% yield and >99% ee (Scheme 6) [19].

### Cross-benzoin reactions

A cross-benzoin reaction unites two different aldehydes wherein one of them functions as the acyl anion equivalent. A total of four products are possible; a pair of homo-benzoin and cross-benzoin adducts each. A substrate-driven selectivity may be observed when one of the aldehydes is significantly less reactive due to electronic or steric reasons. The latter effect may be amplified by employing bulky NHCs. In general, NHC-mediated selective cross-benzoin reactions of electronically and sterically similar aldehydes remain as a highly challenging transformation.

**Scheme 7:**
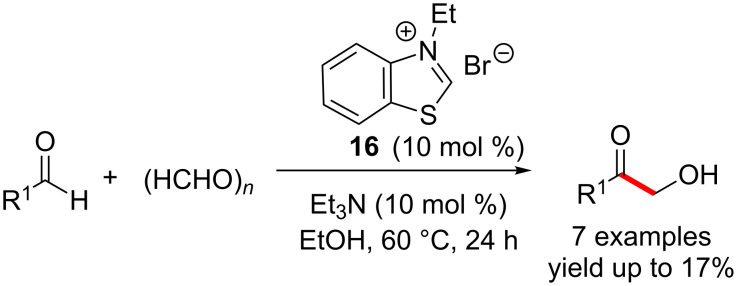
Inoue’s report of cross-benzoin reactions.

In 1985, Inoue and co-workers reported the NHC-catalysed selective cross-benzoin reactions of aromatic and aliphatic aldehydes with formaldehyde leading to the formation of α-hydroxy ketones. Although an excellent selectivity was observed for the cross-benzoin product, the yields were low (Scheme 7) [20].

**Scheme 8:**
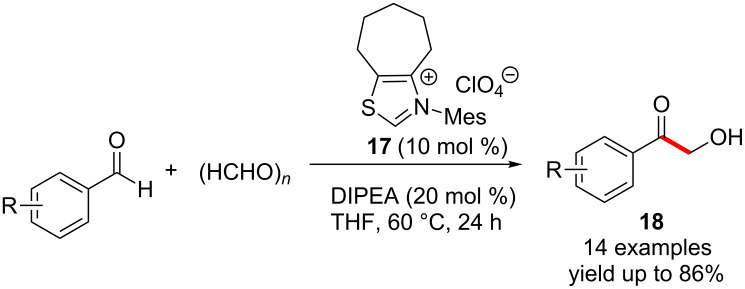
Cross-benzoin reactions catalysed by thiazolium salt **17**.

Later Kuhl and Glorius employed an NHC generated from the thiazolium salt **17** to synthesise α-hydroxyketones **18** in good yields. This highly selective cross-benzoin reaction has a very broad substrate scope (Scheme 8) [21].

**Scheme 9:**
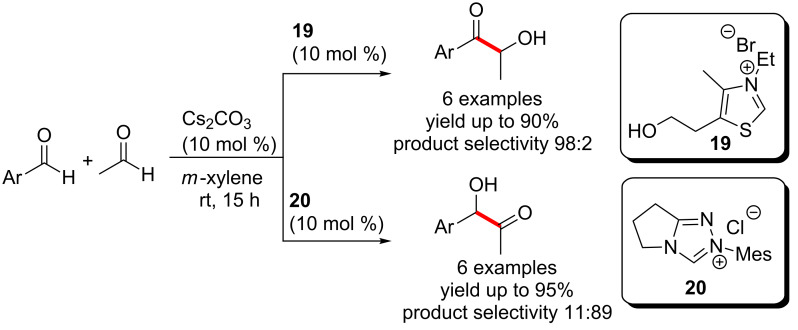
Catalyst-controlled divergence in cross-benzoin reactions.

Yang and co-workers developed an intermolecular cross coupling of aromatic aldehydes with acetaldehyde. The reaction showed an interesting divergence in reactivity controlled by the catalysts, viz., the thiazolium salt **19** and triazolium salt **20**. The thiazolium-derived carbene preferentially mediated the formation of the Breslow intermediate from the aromatic aldehyde followed by coupling with acetaldehyde. In contrast, the triazolium-derived carbene prefered to activate acetaldehyde to generate the corresponding acyl anion equivalent followed by coupling with aromatic aldehydes (Scheme 9) [22]. It may be mentioned that Connon, Zeitler and co-workers have also reported the use of thiazolium and triazolium precatalysts for selective cross-benzoin reactions [23].

**Scheme 10:**
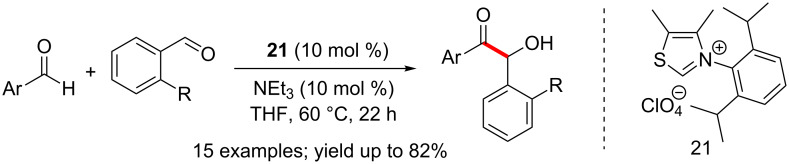
Chemoselective cross-benzoin reactions catalysed by a bulky NHC.

Glorius introduced a number of thiazolium NHC precatalysts endowed with sterically bulky aryl groups on the nitrogen with varying backbone substitution. These NHCs exhibited high levels of reactivity and selectivity in intermolecular cross-benzoin reactions to afford a library of unsymmetrically substituted benzoins [24]. The presence of an *ortho*-substituent on the electrophilic aromatic aldehyde (which presumably hinders the direct addition of NHC to these aldehydes) was necessary for the high levels of selectivity (Scheme 10).

**Scheme 11:**
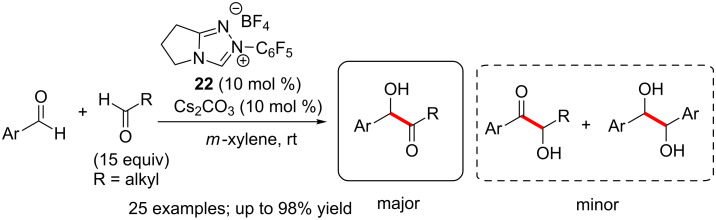
Selective intermolecular cross-benzoin condensation reactions of aromatic and aliphatic aldehydes.

The NHC-catalysed chemoselective intermolecular cross-benzoin condensation reaction of aromatic and aliphatic aldehydes was reported by Yang and co-workers. The chemoselectivity was achieved by using a large excess of the aliphatic aldehyde (molar ratio of 1:15) [25]. Thus, directing groups on the aromatic aldehydes were not a prerequisite for high levels of selectivity in contrast to the earlier example. Consecutive catalytic reactions were utilized in order to reuse the excess of aliphatic aldehydes employed for achieving selectivity. Interestingly, the reaction could be repeated up to five times without affecting the yield of product and chemoselectivity (Scheme 11).

**Scheme 12:**
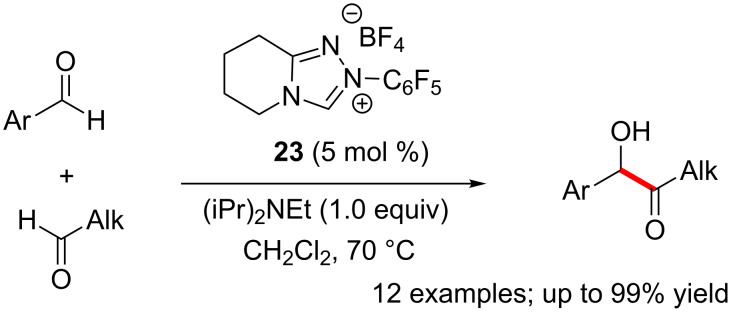
Chemoselective cross-benzoin reaction of aliphatic and aromatic aldehydes.

Morpholinone and piperidinone-derived triazolium precatalysts can catalyze highly chemoselectively the cross-benzoin reaction of aliphatic and aromatic aldehydes [26]. Smooth and selective benzoin reactions were observed with a wide variety of linear and branched aliphatic aldehydes as well as aromatic aldehydes (Scheme 12). Notably, the aliphatic aldehydes functioned as acyl anion equivalents leading to the formation of alkyl ketone (benzoin) products.

### Asymmetric cross-benzoin reactions

The development of enantioselective cross-benzoin reactions is an arduous task as both chemoselectivity and stereoselectivity must be controlled by a single catalyst. Unsurprisingly, most of the NHC-catalysed, enantioselective cross-benzoin reactions employ a combination of two distinct carbonyl components to minimize chemoselectivity issues. A selected group of asymmetric cross-benzoin reactions are described in the following section.

**Scheme 13:**
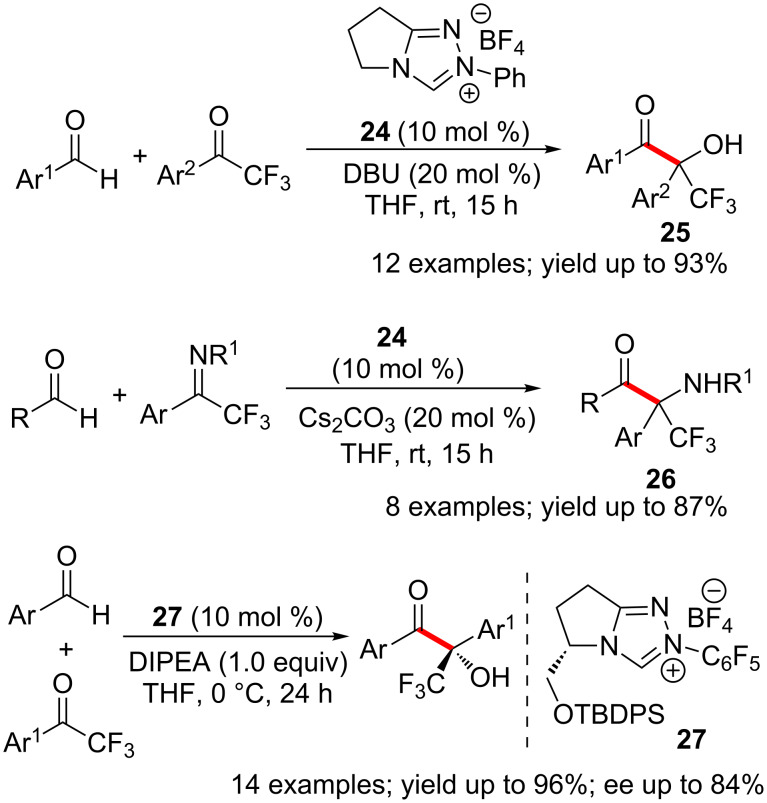
Cross-benzoin reactions of trifluoromethyl ketones developed by Enders.

An NHC-catalysed union of aryl aldehydes and aryl trifluoromethyl ketones was developed in the laboratory of Enders. This direct intermolecular cross-benzoin reaction proceeded with high yields and chemoselectivity [27]. The reaction furnished excellent yields of α-hydroxy-α-trifluoromethyl ketones **25** possessing a quaternary stereocentre. The homo-benzoin condensation between two aldehydes is reversible under the reaction conditions. This eventually leads to the selective formation of the observed cross-benzoin product. Later, it was found that trifluoromethyl ketimines **26** also function as electrophiles under similar reaction conditions [28]. Although initial attempts of asymmetric transformations were not successful, enantioselective cross-benzoin reactions of heteroaromatic aldehydes (acyl donors) and aryl trifluoromethyl ketones were later developed using the chiral catalyst **27** (Scheme 13) [29].

**Scheme 14:**
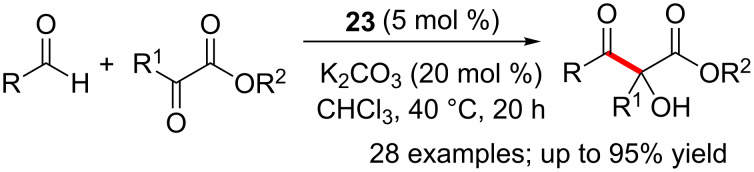
Cross-benzoin reactions of aldehydes and α-ketoesters.

The electron-deficient triazolium-derived NHC **23** mediated efficient and chemoselective cross-benzoin reactions of aldehydes and α-ketoesters to produce acyloin products endowed with a quaternary stereocentre [30]. Remarkably, the competing hydroacylation reaction was not observed under these reaction conditions. A variety of aliphatic and aromatic aldehydes functioned as acyl donors, whereas several α-ketoesters could be employed as the electrophilic coupling partner to afford the desired products in moderate to good yields (Scheme 14). Interestingly, preliminary experiments to develop an enantioselective version of this reaction using a chiral NHC returned promising levels of enantioselectivity (76% ee).

**Scheme 15:**
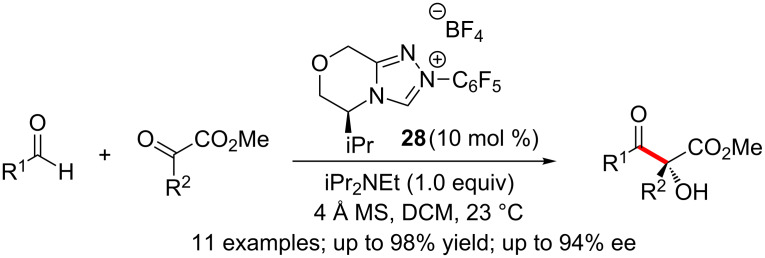
Enantioselective cross-benzoin reactions of aliphatic aldehydes and α-ketoesters.

Subsequently, Gravel and co-workers reported a high yielding chemoselective and enantioselective intermolecular cross-benzoin reaction of aliphatic aldehydes and α-ketoesters. Notably, the reaction affords enantiomerically enriched tertiary alcohols. Excellent levels of enantioselection were obtained by using an electron-deficient valine-derived triazolium salt precatalyst **28** (Scheme 15) [31]. Moreover, diastereoselective reduction of the cross-benzoin products with NaBH_4_ afforded valuable *syn*-diol products.

**Scheme 16:**
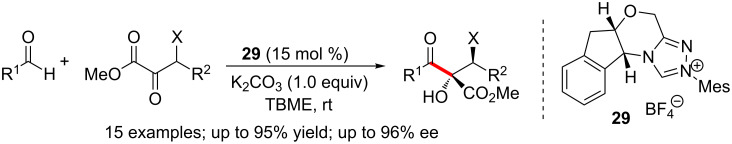
Dynamic kinetic resolution of β-halo-α-ketoesters via cross-benzoin reaction.

Goodman and Johnson disclosed a dynamic kinetic resolution of β-halo-α-ketoesters via NHC-catalysed asymmetric cross-benzoin reaction. Here, the cross-benzoin reaction of aromatic aldehydes with β-stereogenic-α-keto esters afforded fully substituted β-halo-α-glycolic acid derivatives in high diastereoselectivity and enantioselectivity [32]. The NHC generated from the amino indanol-derived chiral triazolium salt **29** provided the best results (Scheme 16). A variety of aromatic aldehydes and a series of β-halo α-ketoesters partake in the reaction to furnish the chiral glycolic acid derivatives.

**Scheme 17:**
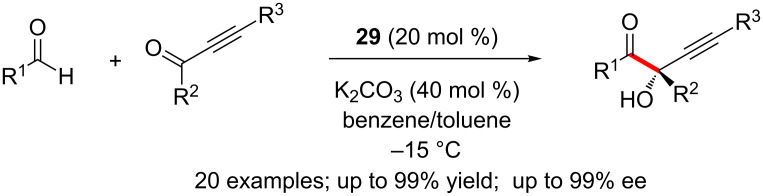
Enantioselective benzoin reaction of aldehydes and alkynones.

The enantioselective benzoin reaction between a variety of aldehydes and alkynones is catalysed by the NHC generated from chiral aminoindanol-triazolium salt **29**. The reactions afforded substituted propargylic alcohols in high yields and enantioselectivity (Scheme 17). It is noteworthy that the catalytically generated Breslow intermediates undergo selective 1,2-addition with ynones and the competing Stetter-type reactivity was not observed [33].

### Aza-benzoin reactions

In aza-benzoin reactions, the acyl anions generated from aldehydes react with an aza electrophile. Imines possessing an electron-withdrawing N-substituent constitute the most commonly used aza electrophile and the reaction affords an α-aminocarbonyl compound as the product. The NHC-mediated addition of aldehyde-derived acyl anions to nitroso compounds leading to the formation of hydroxamic acid derivatives are also discussed in this section for convenience.

**Scheme 18:**
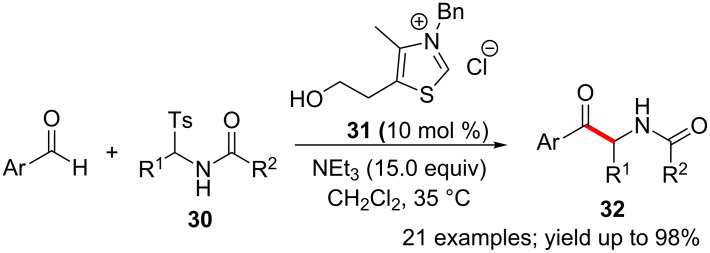
Aza-benzoin reaction of aldehydes and acylimines.

Acylimines function as electrophiles in NHC-catalysed aza-benzoin reaction with aldehydes. The reactive acylimine is generated in situ by the action of base on the sulfonylamide derivative **30** [34]. Meanwhile, the Breslow intermediate is produced from the aldehyde by the thiazolium **31**-derived NHC. The union of these two reactive intermediates furnished α-amidoketones **32** in excellent yields (Scheme 18).

**Scheme 19:**
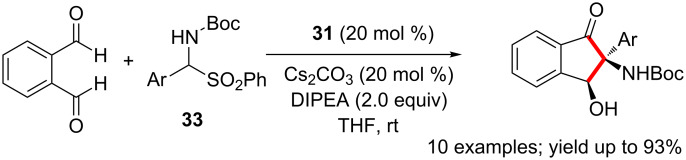
NHC-catalysed diastereoselective synthesis of *cis*-2-amino 3-hydroxyindanones.

A diastereoselective [4 + 1] annulation of phthalaldehyde with imines leading to the formation of *cis*-2-amino3-hydroxyindanones is catalysed by NHC **31**. The imine electrophile is generated in situ from α-sulfonyl-*N*-Boc amine **33** (Scheme 19). Initial cross-aza-benzoin reaction of one of the aldehyde functionalities with the imine is followed by an intramolecular aldol reaction to furnish the indanone framework [35].

**Scheme 20:**
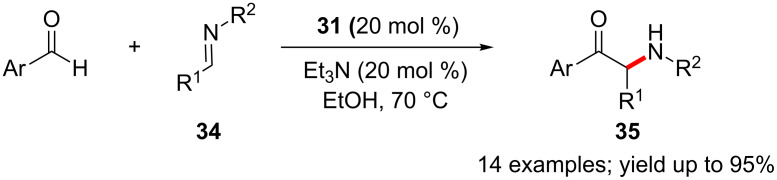
Cross-aza-benzoin reactions of aldehydes with aromatic imines.

The thiazolium precatalyst **31** can also efficiently mediate cross-aza-benzoin reactions of aromatic and heteroaromatic aldehydes with unactivated aromatic imines **34** (Scheme 20) [36]. A control reaction of the corresponding benzoin (instead of the aldehyde) and imine **34** also afforded the α-amino ketone product **35** in 71% yield. This indicates that the reaction involves reversible formation of aldehyde-homobenzoin adducts.

**Scheme 21:**
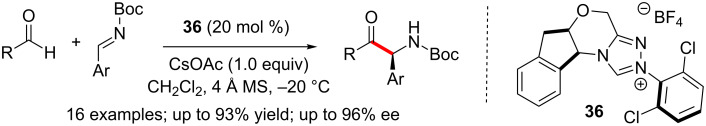
Enantioselective cross aza-benzoin reaction of aliphatic aldehydes with *N*-Boc-imines.

Enantioselective cross aza-benzoin reaction of aliphatic aldehydes with *N*-Boc-protected imines are promoted efficiently by NHC generated from the chiral triazolium salt **36**. The aldehydes function as the acyl donor and the imines behave as the receptors (Scheme 21). Addition of NHC to the highly electrophilic *N*-Boc imines leads to the formation of corresponding aza-Breslow intermediates; however, it is reversible under the reaction conditions. Importantly, the chirally pure α-amino ketones formed in this reaction are valuable building blocks in organic synthesis [37].

**Scheme 22:**
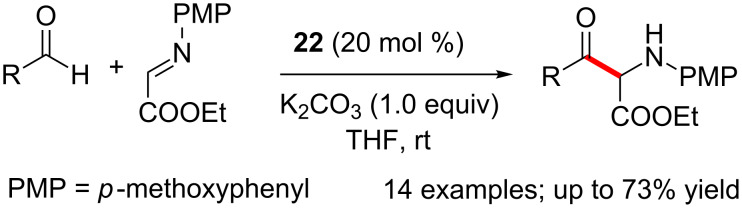
Chemoselective cross aza-benzoin reaction of aldehydes with *N*-PMP-imino esters.

The NHC generated from the bicyclic pentafluoro triazolium salt promoted the chemoselective cross aza-benzoin reaction of aldehydes with *N*-PMP-imino esters to afford α-amino-β-keto esters in good yield (Scheme 22) [38]. A range of functional groups are tolerated under the optimised reaction conditions.

**Scheme 23:**
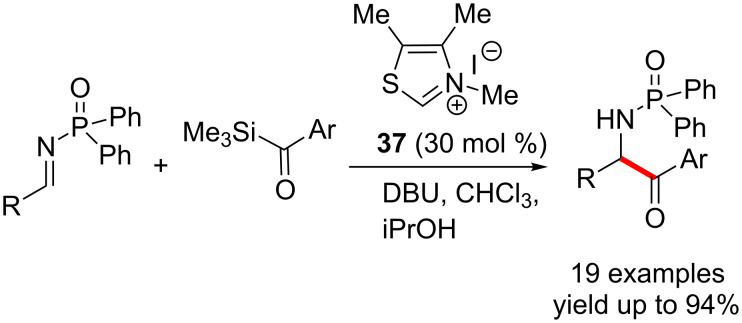
NHC-catalysed coupling reaction of acylsilanes with imines.

Mattson and Scheidt developed a catalytic coupling reaction of acylsilanes with imines for the synthesis of aminoketones (Scheme 23). The reaction proceeds through the generation of the Breslow intermediate from the acylsilane followed by a cross-coupling with the imine [39].

**Scheme 24:**
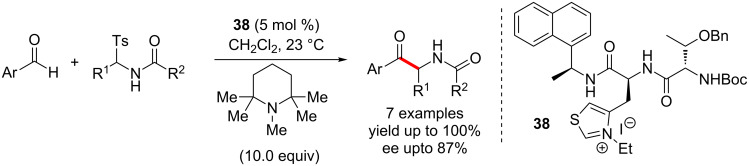
Thiazolium salt**-**mediated enantioselective cross-aza-benzoin reaction.

In 2005, Miller and co-workers used the chiral thiazolium salt **38** to catalyse an enantioselective cross-aza-benzoin reaction. Racemisation of the products under the reaction conditions caused erosion of enantioselectivity. This problem was successfully addressed by using a hindered base, pentamethyl piperidine, which was inert towards the products (Scheme 24) [40].

**Scheme 25:**
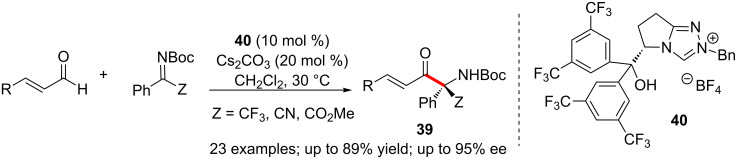
Aza-benzoin reaction of enals with activated ketimines.

In 2013, Ye disclosed a remarkable NHC-catalysed enantioselective aza-benzoin reaction of enals and activated ketimines leading to the formation of functionalised α-aminoketones **39** in high enantioselectivity [41]. Notably, the homoenolate or enolate reactivity of the NHC-enal adduct was not observed in this case. The presence of a tertiary alcohol functionality and the steric bulk of the NHC-precatalyst **40** were essential for the selective formation of the aza-benzoin adduct. A variety of trifluoromethylated α-aminoketones could be synthesised in enantiomerically pure form using this method (Scheme 25).

**Scheme 26:**
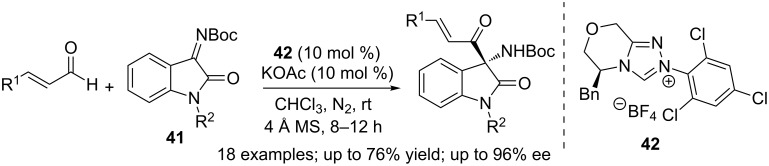
Isatin derived ketimines as electrophiles in cross aza-benzoin reaction with enals.

Isatin derived ketimines **41** were employed as electrophiles in the NHC-catalysed chemo- and stereoselective cross-aza-benzoin reaction with enals by Chi. The reaction afforded chiral quaternary aminooxindole derivatives. The NHC–enal adduct prefers to react via the acyl anion pathway and the competing homoenolate/enolate reactivity was not observed. The sterically non-congested, electron-deficient NHC-catalyst **42** presumably does not hinder bond formation at the catalyst-bound acyl carbon (Scheme 26) [42].

**Scheme 27:**
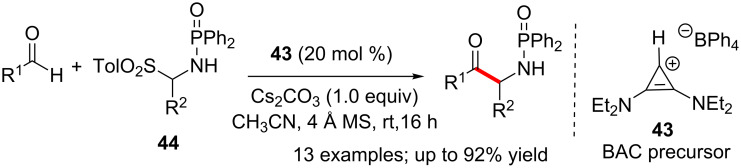
Aza-benzoin reaction of aldehydes and phosphinoylimines catalysed by the BAC-carbene.

The aza-benzoin reaction of aldehydes and phosphinoylimines catalysed by the bis(amino)cyclopropenylidene (BAC) carbene **43** was reported recently. The reaction showed excellent selectivity for the aza-benzoin products over the homo-benzoin adducts. A wide variety of aldehydes react with phosphinoylimines (generated from their sulfinic acid adducts **44**) to afford *N*-phosphinoyl amnioketones (Scheme 27) [43]. The attempted enantioselective version of this reaction using a chiral BAC catalyst was, however, unsuccessful.

**Scheme 28:**
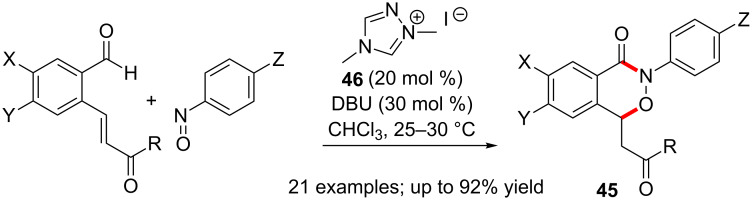
Nitrosoarenes as the electrophilic component in benzoin-initiated cascade reaction.

As mentioned earlier, nitrosoarenes have been used as the electrophilic component in a few reactions of NHC-bound aldehydes. The addition of acyl anions occur at the nitrogen atom of the nitroso compound. A NHC-catalysed cascade reaction of *o*-vinylarylaldehydes with nitrosoarenes afforded functionalised 2,3-benzoxazin-4-ones **45** [44]. The initial intermolecular aza-benzoin reaction is followed by an intramolecular oxa-Michael reaction to form the observed product (Scheme 28).

**Scheme 29:**
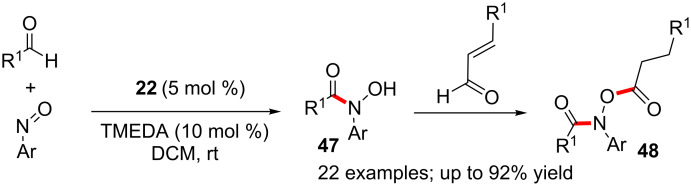
One-pot synthesis of hydroxamic esters via aza-benzoin reaction.

Enders reported a cascade reaction which is initiated by an NHC-catalysed aza-benzoin condensation between various aldehydes and nitrosobenzenes to generate the hydroxamic acids **47**. This is followed by a redox esterification of the latter (**47**) with enals. The overall process constitutes a one-pot synthesis of hydroxamic esters **48** [45]. Notably, both steps can be performed using the single NHC catalyst **22** under same reaction conditions (Scheme 29). This two-step, one-pot synthesis of formahydroxamic esters constitutes a valuable addition to a thin list of NHC-mediated three-component reactions.

### Intramolecular benzoin reactions

**Scheme 30:**
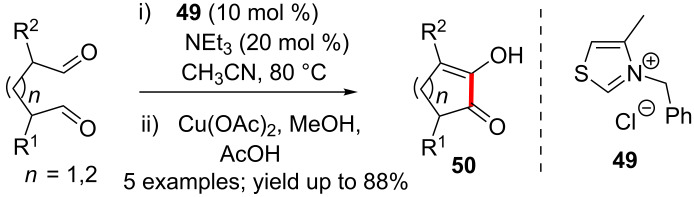
Cookson and Lane’s report of intramolecular benzoin condensation.

One of the earliest reports of an intramolecular benzoin condensation appeared in 1976. Cookson and Lane found that the treatment of anhydrous glutaraldehyde with thiazolium salt **49** and triethylamine resulted in the formation of 2-hydroxycyclopentanone. The latter underwent oxidation to afford 2-hydroxycyclopent-2-en-1-one **50** upon treatment with Cu(OAc)_2_ (Scheme 30) [46]. Hexanedial furnished the corresponding α-hydroxycyclohexanone under identical reaction conditions.

**Scheme 31:**
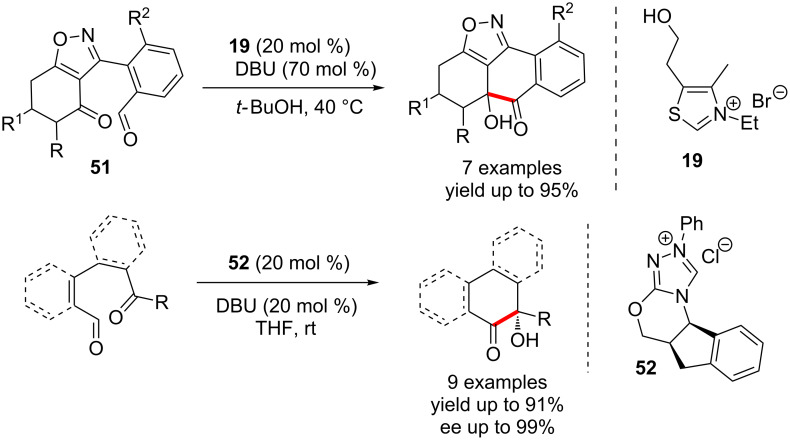
Intramolecular cross-benzoin condensation between aldehyde and ketone moieties.

An intramolecular cross-benzoin condensation between aldehyde and ketone moieties was developed by Suzuki in 2003. The isoxazole-fused cyclohexanone **51** endowed with an aryl aldehyde underwent a smooth cross-benzoin cyclisation in the presence of the thiazolium catalyst **19** and DBU. Although the presence of an isoxazole moiety is not a prerequisite for the success of this annulation, its rigid nature presumably renders the reaction highly stereoselective [47]. This simple and mild method allowed the construction of orthogonally protected polycyclic quinones from readily available starting materials. Later in 2006, they developed the enantioselective version of this reaction using an aminoindanol-derived triazolium salt **52** (Scheme 31) [48].

**Scheme 32:**
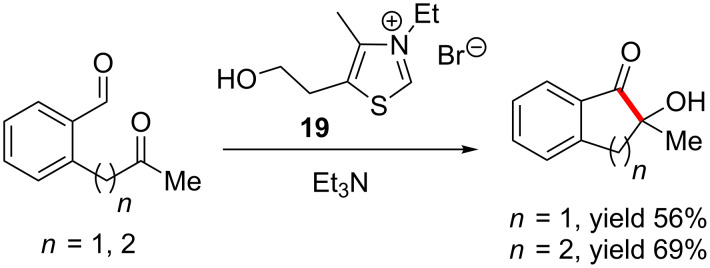
Intramolecular crossed aldehyde-ketone benzoin reactions.

Another intramolecular crossed aldehyde-ketone benzoin reaction of simple dicarbonyl systems was developed by Enders (Scheme 32). This method employs commercially available thiazolium salt **19** as precatalyst and affords five- and six-membered cyclic acyloins in good yields [49].

**Scheme 33:**
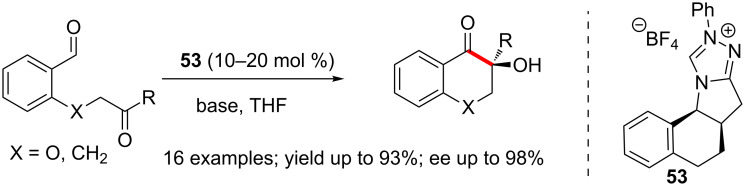
Enantioselective intramolecular crossed aldehyde-ketone benzoin reaction.

The enantioselective NHC-catalysed crossed aldehyde-ketone benzoin reaction for the synthesis of five- and six-membered cyclic acyloins was also developed by Enders. NHC generated from the tetracyclic triazolium salt **53** gave the best results [50]. It is noteworthy that the absolute stereochemistry of the α-carbonyl quaternary center of benzo-fused carbocycles and chromanones is installed with excellent control (Scheme 33).

**Scheme 34:**
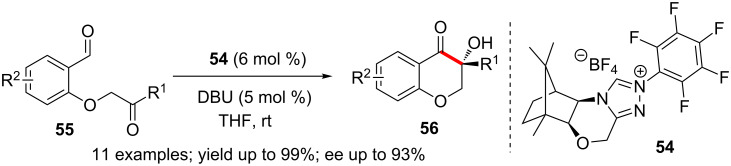
Chromanone synthesis via enantioselective intramolecular cross-benzoin reaction.

A combination of D-camphor-derived triazolium precatalyst **54** and DBU promoted enantioselective intramolecular cross-benzoin reaction of **55** to afford chromanone **56** in excellent yield and enantioselectivity (Scheme 34). The NHC-precatalyst is conveniently prepared from camphor in 5 steps [51].

**Scheme 35:**
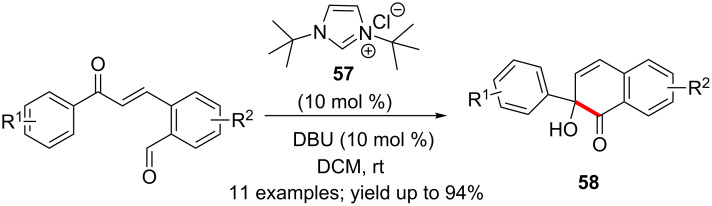
Intramolecular cross-benzoin reaction of chalcones.

NHC generated from the *N*-*tert*-butyl-substituted imidazolium salt **57** catalysed the intramolecular cross-benzoin reaction of chalcones derived from *o*-phthalaldehydes. The reaction proceeded rapidly (20 min) at room temperature to afford good yields (75–94%) of naphthalenone-based tertiary alcohols **58** (Scheme 35) [52].

**Scheme 36:**
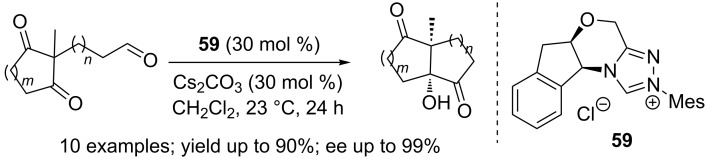
Synthesis of bicyclic tertiary alcohols by intramolecular benzoin reaction.

The synthesis of bicyclic tertiary alcohols possessing two quaternary stereocentres at the bridgehead positions was achieved via an asymmetric intramolecular crossed benzoin reaction. A relatively high loading (30 mol %) of the NHC precatalyst **59** was necessary for efficient reactions (Scheme 36) [53].

**Scheme 37:**
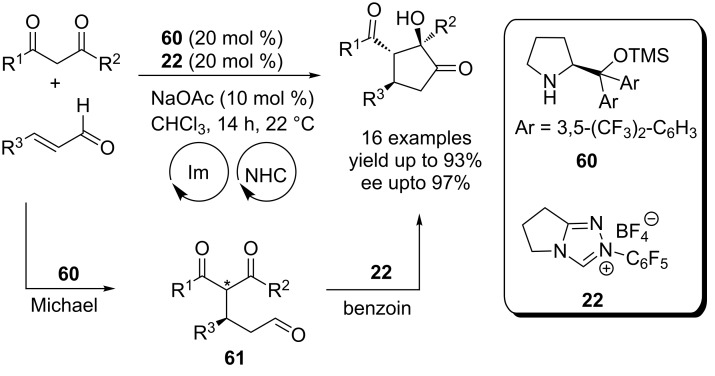
A multicatalytic Michael–benzoin cascade process for cyclopentanone synthesis.

A multicatalytic Michael–benzoin cascade process for the asymmetric synthesis of functionalised cyclopentanones was disclosed in 2009 by Rovis. The chiral secondary amine **60** catalyzes the initial asymmetric Michael addition of an 1,3-diketone and an enal to afford a δ-ketoaldehyde **61**. Subsequently, a cross-benzoin reaction of the latter promoted by the NHC precatalyst **22**, installs the cyclopentenone system (Scheme 37). It may be noted that the absolute stereochemistry of the process is controlled by the prolinol catalyst **60** and the NHC precatalyst **22** is achiral. Control experiments revealed that the Michael addition is reversible but the NHC catalyst rapidly shuttles the intermediate δ-keto aldehyde **61** to the final product preventing the erosion of enantioselectivity [54]. This cascade reaction constitutes a fine example of symbiotic dual-catalysis wherein both catalysts perform better together in a one-pot reaction than they do independently over two steps.

**Scheme 38:**
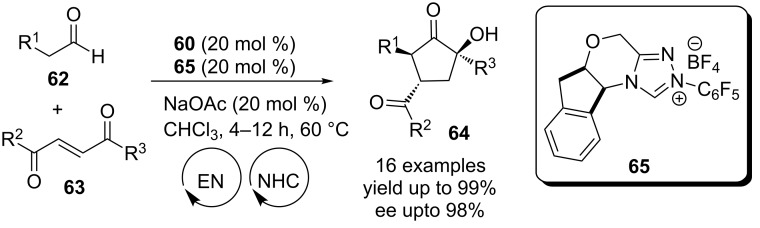
Enamine-NHC dual-catalytic, Michael–benzoin cascade reaction.

A conceptually similar enamine-NHC dual-catalytic Michael–benzoin cascade was also developed by Rovis. The reaction proceeds via the generation of an enamine from the enolizable aldehyde **62** in presence of the prolinol catalyst **60** and its subsequent addition to the Michael acceptor **63**. This is followed by NHC-mediated intramolecular cross-benzoin condensation to afford the cyclopentanone **64**. Clear evidence for the co-operative relationship between the catalysts was obtained from control experiments. Chiral triazolium catalyst **65** preferentially converts only one of the diastereomeric Michael adducts into the benzoin product. The prolinol catalyst **60**, on the other hand, mediates the epimerisation of the less reactive diastereomer. This synergy leads to the enrichment of the diastereomeric ratio of the final product **64** (Scheme 38) [55].

**Scheme 39:**
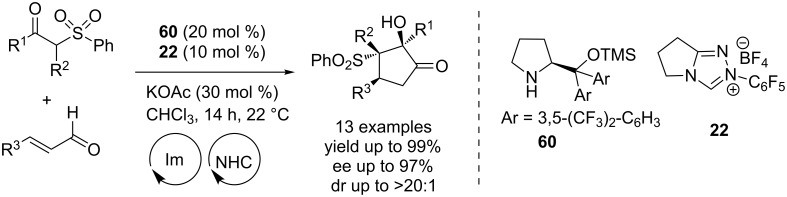
Iminium-cross-benzoin cascade reaction of enals and β-oxo sulfones.

Enders developed a closely related iminium-cross-benzoin cascade process involving enals and β-oxo sulfones to generate enantioenriched cyclopentanone derivatives with three contiguous stereocentres. A dual secondary amine/NHC catalytic system comprising of the prolinol **60** and NHC precatalyst **22** was found to give the best results (Scheme 39) [56]. The influence of these catalysts on the diastereoselectivity of the reaction was also studied using NMR techniques.

**Scheme 40:**
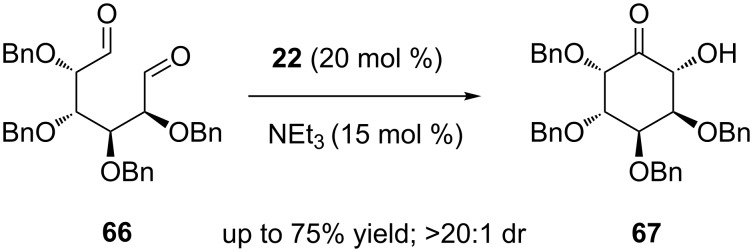
Intramolecular benzoin condensation of carbohydrate-derived dialdehydes.

An NHC-catalysed intramolecular benzoin condensation of carbohydrate-derived dialdehydes has been applied for the construction of carbocyclic sugars. Diastereoselective benzoin reactions of manno- and galacto-configured dialdehydes **66** were promoted by the triazolium carbene precatalyst **22** to produce single inosose stereoisomers **67** in high yields (Scheme 40) [57]. Stereospecific reduction and deprotection of the inosose derivatives furnished *allo*- and *epi*-inositol in good yields.

**Scheme 41:**
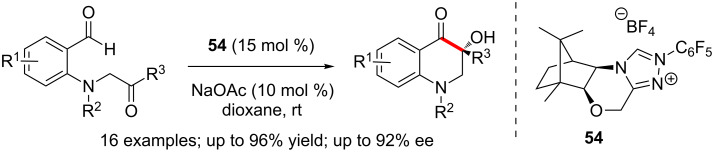
Enantioselective intramolecular benzoin reactions of *N*-tethered keto-aldehydes.

The camphor-derived triazolium precatalyst **54** promoted enantioselective intramolecular benzoin reactions of *N*-tethered keto-aldehydes effectively. The substrates for the cyclisation are easily accessible and dihydroquinolinone systems possessing a quaternary stereocentre are produced in high yields and enantioselectivities (Scheme 41) [58].

**Scheme 42:**
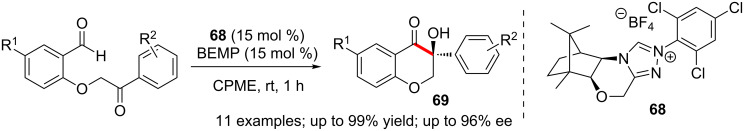
Asymmetric cross-benzoin reactions promoted by camphor-derived catalysts.

The chiral triazolium salt **68** derived from (1*R*)-camphor has been used in intramolecular cross-benzoin reactions of keto-aldehydes. The former efficiently catalysed stereoselective formation of chromanones **69** bearing quaternary stereocentres (Scheme 42) [59].

**Scheme 43:**
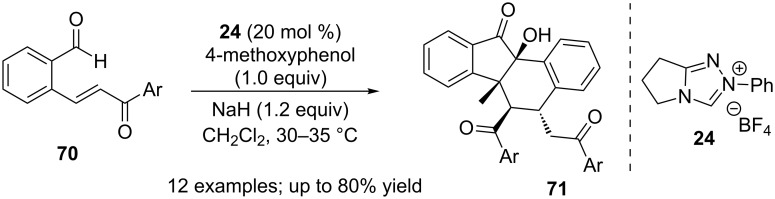
NHC-Brønsted base co-catalysis in a benzoin–Michael–Michael cascade.

Cheng reported that the combination of NHC **24** and a Brønsted base (4-methoxyphenolate) promoted a formal dimerisation of 2-(aroylvinyl)arylaldehydes **70** to afford benzo[*a*]tetrahydrofluorenones **71** [60]. This stereoselective reaction proceeds via a benzoin–Michael–Michael cascade process (Scheme 43).

**Scheme 44:**
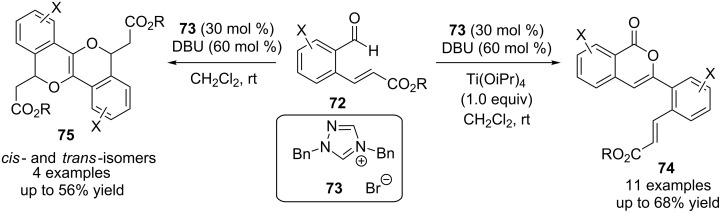
Divergent catalytic dimerization of 2-formylcinnamates.

Further investigations in Cheng’s group revealed an intriguing divergent catalytic dimerisation of 2-formylcinnamates **72**. Co-operative catalysis by NHC precatalyst **73** and a Lewis acid (titanium isopropoxide) afforded isochromenone derivatives **74** via a sequence of reactions initiated by a benzoin condensation. Treatment of **72** with the NHC precatalyst **73** alone, on the other hand, afforded isochromeno(4,3-*c*)isochromene derivatives **75** (Scheme 44) [61].

**Scheme 45:**
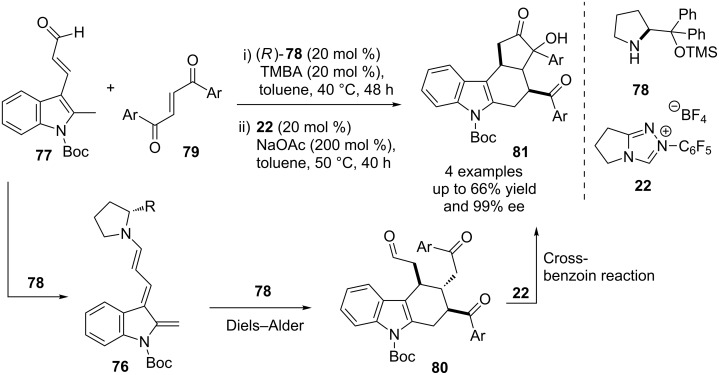
One-pot, multicatalytic asymmetric synthesis of tetrahydrocarbazole derivatives.

A one-pot multicatalytic reaction for the asymmetric synthesis of complex tetracyclic tetrahydrocarbazole derivatives from readily available precursors was described by Melchiorre. A Diels–Alder reaction of indole-2,3-quinodimethane **76** (generated from **77** and the prolinol catalyst **78** ) with the enone **79** affords a tetrahydrocarbazole derivative **80**. The NHC precatalyst **22** then promotes an intramolecular cross-benzoin condensation of the keto-aldehyde to furnish the tetracyclic product **81** (Scheme 45). The yields are moderate; however excellent diastereo- and enantioselectivities were observed for the one-pot reaction [62].

**Scheme 46:**
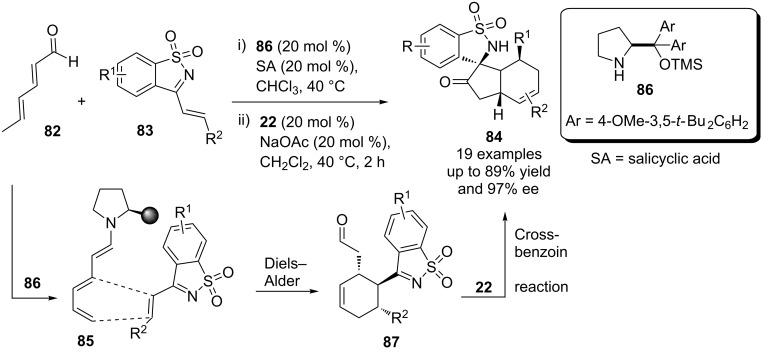
NHC-chiral secondary amine co-catalysis for the synthesis of complex spirocyclic scaffolds.

In a similar fashion, an asymmetric multicatalytic cascade reaction involving the dienal **82** and unsaturated cyclic sulfonylimine **83** afforded spiro-fused cycloadducts **84** in good yield and enantioselectivity [63]. Initially, the trienamine **85** is generated by the action of prolinol catalyst **86** on the dienal **82**. The former (**85**) then undergoes a Diels–Alder reaction with the sulfonylimine **83** to generate the keto-aldehyde **87**. Finally, the NHC precatalyst **22** mediates a cross-benzoin reaction of the latter to furnish the spirocyclic product **84** (Scheme 46).

## Conclusion

The first report of a benzoin reaction by Wöhler and Liebig appeared merely four years after the former disclosed the paradigm-changing urea synthesis. However, detailed investigations of this reaction remained elusive due to a variety of reasons, the toxicity of cyanide catalysts being one of them. Breslow’s discovery in 1958 of the thiazolylidene-catalysed benzoin condensation via polarity reversal of aldehydes formed the conceptual basis for the later development of NHC-organocatalysis. The rekindling of interest in NHC-catalysed benzoin reactions coincided with the emergence of N-heterocyclic carbenes in the late twentieth century as non-toxic, readily available and versatile catalysts for a variety of organic transformations. Since then, a number of reports on a variety of benzoin reactions have appeared in the literature. They include homo, crossed, intramolecular and various asymmetric benzoin reactions leading to products that are difficult to access by other means. Aza-benzoin reactions, intramolecular benzoin condensations, use of aldehyde surrogates and use of non-carbonyl electrophiles (nitroso compounds) are some of the developments that revamped the synthetically unattractive, monotonous image of benzoin condensations. The driving force behind this remarkable evolution of benzoin reaction is NHC-catalysis. Benzoin chemistry is well-set to benefit, in the near future, from new developments in the rapidly growing realm of NHC-catalysis.
