# Correction: Recent advances in N-heterocyclic carbene (NHC)-catalysed benzoin reactions

**DOI:** 10.3762/bjoc.12.201

**Published:** 2016-10-04

**Authors:** Rajeev S Menon, Akkattu T Biju, Vijay Nair

**Affiliations:** 1 Department of Chemistry, Central University of Haryana, Mahendergarh, Haryana-123 029, India; 2 Organic Chemistry Division, National Chemical Laboratory (CSIR), Dr. Homi Bhabha Road, Pune 411 008, India; 3 Organic Chemistry Section, CSIR-National Institute for Interdisciplinary Science and Technology,Trivandrum 695 019, India.; Fax: +91 471 2491712; Tel: +91 471 2490406

**Keywords:** acyloin reaction, benzoin reaction, N-heterocyclic carbenes, organocatalysis, umpolung

On page 446, column 2, the sentence “Inoue and co-workers found that it promotes homocoupling of benzaldehyde at a low loading (4 mol %) to afford benzoin in 90% yield and >99% ee (Scheme 6) [19]” should be read as “Zeitler and Connon reported that it promotes homocoupling of benzaldehyde at a low loading (4 mol %) to afford benzoin in 90% yield and >99% ee (Scheme 6) [19].”

The oversight, and the consequent inconvenience to Professor Zeitler and Professor Connon and their co-workers, as well as the readers of the *Beilstein Journal of Organic Chemistry* is highly regretted.

